# Immune Cells Strike a Balance to Avoid Autoimmune Disease

**DOI:** 10.1371/journal.pbio.0040393

**Published:** 2006-10-17

**Authors:** Mason Inman

Immune cells walk a fine line, biochemically speaking. They must be sensitive enough to recognize foreign invaders like bacteria and viruses, but not so reactive that they attack an animal’s own tissues, paving the way for autoimmune disorders. Animals use a variety of mechanisms to strike this balance. One way is by regulating the activity of T cells—immune cells that can trigger an immune response to billions of different molecules—through a range of processes. In one process known as deletion, autoreactive T cells (which attack the body) are killed off. Another process involves making a subset of T cells develop into a specialized type, called suppressor T cells (or T regulatory cells). The suppressor T cells can then regulate the behavior of other immune cells, damping down autoimmune reactions.

In addition to these two well-studied mechanisms for avoiding autoimmunity, evidence is mounting that animals have another tool at their disposal, a process called adaptation. This process seems to come into play especially when the immune system faces a persistent infection or continually reacts to the body’s own tissues. In these cases, avoiding a fatal autoimmune response requires keeping the immune system in check. In adaptation, immune cells become habituated to foreign (or apparently foreign) proteins and tone down their activity. But how adaptation works together with deletion and regulation by suppressor T cells to avoid autoimmunity has been difficult to tease out.

Nevil Singh, Chuan Chen, and Ronald Schwartz studied adaptation in isolation from the other mechanisms for avoiding autoimmunity and found support for the view that adaptation is a mechanism independent of either deletion or regulation by suppressor T cells. The researchers injected T cells into a strain of mice that were engineered to lack their own T cells. The injected T cells hadn’t yet encountered any foreign or activating proteins (and so are called “naive” T cells). Singh and colleagues could watch the injected T cells adjust to their new environment, and because the host animals had no T cells of their own, the naive T cells could adjust without interference from native T cells that had matured in the mice’s bodies.

The researchers found that the injected T cells multiplied, as they do in mounting an immune response to something foreign, but then over about a week, adapted to the new environment. The injected T cells toned down their activity, but persisted in the animals’ bodies for more than two months (without invoking deletion). They did not develop into suppressor T cells, either.

**Figure:**
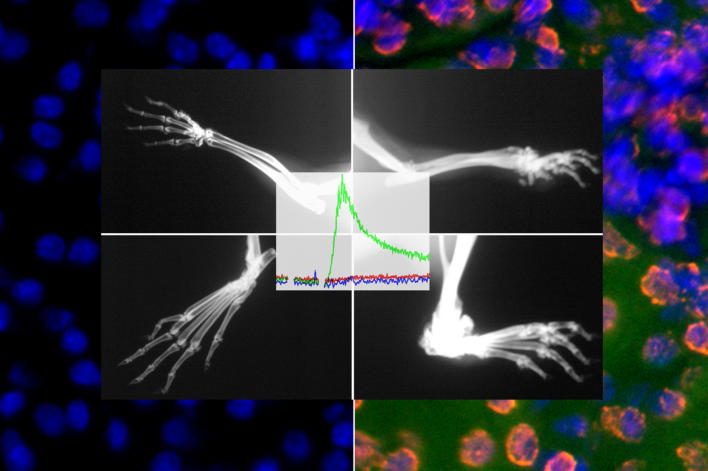
Self-reactivity of T cells can be down-regulated by cell-intrinsic adaptation (as indicated by reduced calcium ? uxes of adapted T cells shown in the center inset). Left and right panels show samples from mice with and without autoreactive T cells, respectively. (Image: Nevil Singh et al.)

Despite the adaptation of the injected T cells, they caused the mice to develop arthritis, an autoimmune disease, after a few months. This was a surprise, since the T cells appeared to have adjusted to being in the mice. To pin down how the T cells caused this autoimmune response, the researchers injected naive T cells into another mouse strain, which lacks B cells—immune cells that create antibodies against foreign proteins. The B cells typically do this only after being triggered by T cells that have recognized foreign cells. The researchers let the T cells adapt to being in the mice, and then introduced B cells from outside. Surprisingly, although the adapted T cells seemed to tolerate the body’s tissues, they still retained the ability to activate B cells, which then produced a variety of antibodies against the body’s own tissues, eventually leading to arthritis.

In a final set of experiments, Singh and colleagues put the naive T cells into adult mice of a strain born supplied with their own T cells. In this case, contrary to the earlier experiments, the naive T cells did not give the mice arthritis or produce other signs of an autoimmune response. The T cells still undergo adaptation but are subsequently deleted slowly. Interestingly, in the presence of a full set of T cells, the autoreactive T cells also developed fewer effector functions.

Together, these results suggest that to keep T cells from developing in a way that promotes autoimmunity, two kinds of regulation are necessary. First, there’s the adaptation of the T cells to other cells that they see for a long time. And second, there’s the regulation that comes with competing among a variety of T cells that have gone through normal development in the animal. The authors make the case that these different types of regulation have to be understood separately, making a step toward better understanding how autoimmune disorders arise.

